# Cost-effectiveness of adjuvant systemic therapies for patients with high-risk melanoma in Europe: a model-based economic evaluation

**DOI:** 10.1016/j.esmoop.2021.100303

**Published:** 2021-11-13

**Authors:** E.E.A.P. Mulder, L. Smit, D.J. Grünhagen, C. Verhoef, S. Sleijfer, A.A.M. van der Veldt, C.A. Uyl-de Groot

**Affiliations:** 1 Department of Surgical Oncology, Erasmus MC Cancer Institute, Rotterdam, the Netherlands; 2 Department of Medical Oncology, Erasmus MC Cancer Institute, Rotterdam, the Netherlands; 3 Erasmus School of Health Policy & Management, Erasmus University Rotterdam, Rotterdam, the Netherlands; 4 Department of Radiology & Nuclear Medicine, Erasmus MC Cancer Institute, Rotterdam, the Netherlands; 5 Institute for Medical Technology Assessment, Erasmus University Rotterdam, Rotterdam, the Netherlands

**Keywords:** melanoma, adjuvant treatment, pembrolizumab, nivolumab, dabrafenib-trametinib, cost-effectiveness

## Abstract

**Background:**

The introduction of adjuvant systemic treatment has significantly improved recurrence-free survival in patients with resectable high-risk melanoma. Adjuvant treatment with immune checkpoint inhibitors and targeted therapy, however, substantially impacts health care budgets, while the number of patients with melanoma who are treated in the adjuvant setting is still increasing. To evaluate the socioeconomic impact of the three adjuvant treatments, a cost-effectiveness analysis (CEA) was carried out.

**Materials and methods:**

Data were obtained from the three pivotal registration phase III clinical trials on the adjuvant treatment of patients with resected high-risk stage III in melanoma (KEYNOTE-054, CheckMate 238, and COMBI-AD). For this CEA, a Markov model with three health states (no evidence of disease, recurrent/progressive disease, and death) was applied. From a societal perspective, different adjuvant strategies were compared according to total costs, life years (LYs), quality-adjusted life years (QALYs), and incremental cost-effectiveness ratios. To evaluate model uncertainty, sensitivity analyses (deterministic and probabilistic) were carried out.

**Results:**

In the adjuvant setting, total costs (per patient) were €168 826 for nivolumab, €194 529 for pembrolizumab, and €211 110 for dabrafenib-trametinib. These costs were mainly determined by drug acquisition costs, whereas routine surveillance costs varied from €126 096 to €134 945. Compared with routine surveillance, LYs improved by approximately 1.41 for all therapies and QALYs improved by 2.02 for immune checkpoint inhibitors and 2.03 for targeted therapy. This resulted in incremental cost-effectiveness ratios of €21 153 (nivolumab), €33 878 (pembrolizumab), and €37 520 (dabrafenib-trametinib) per QALY gained.

**Conclusions:**

This CEA compared the three EMA-approved adjuvant systemic therapies for resected stage III melanoma. Adjuvant treatment with nivolumab was the most cost-effective, followed by pembrolizumab. Combination therapy with dabrafenib-trametinib was the least cost-effective. With the increasing number of patients with high-risk melanoma who will be treated with adjuvant treatment, there is an urgent need to reduce drug costs while developing better prognostic and predictive tools to identify patients who will benefit from adjuvant treatment.

## Highlights

• Adjuvant treatment with pembrolizumab, nivolumab, or dabrafenib-trametinib improves recurrence-free survival.
• Cost-effectiveness analysis of adjuvant systemic therapies versus routine surveillance in high-risk stage III melanoma.
• Per QALY gained, nivolumab was the most cost-effective (€21 153) and dabrafenib-trametinib the least (€37 520).
• Drug acquisition costs place a significant burden on the health care budget, indicating an urgent need to reduce drug costs.

## Introduction

Cutaneous melanoma is the most aggressive skin cancer and its incidence is increasing worldwide.^1^^,^^2^ Life expectancy of patients with melanoma is determined by disease stage, with 10-year overall survival (OS) rates ranging from 98% for stage I to 32% for stage IV melanoma.^3^^,^^4^ The survival rates of patients with irresectable stage III and metastatic stage IV melanoma have significantly improved since the introduction of systemic therapy with immune checkpoint inhibitors (ICIs) and targeted therapy (TT).^5^, ^6^, ^7^, ^8^ To enhance antitumor T-cell-mediated immune responses, ICIs block programmed cell death protein 1 (PD-1) (nivolumab or pembrolizumab) or cytotoxic T-lymphocyte-associated antigen 4 (CTLA-4) (ipilimumab). Based on their similar mechanism of action and efficacy, nivolumab and pembrolizumab are considered interchangeable.^9^, ^10^, ^11^, ^12^ TTs have a different mechanism of action,^13^ as the selective BRAF inhibitors (e.g. dabrafenib) and MEK inhibitors (e.g. trametinib) block cancer cell proliferation in melanomas driven by a mutated *BRAF* gene (*BRAF*-mt), which is present in 40%-60% of melanomas.^14^^,^^15^

More recently, adjuvant systemic therapy has been shown to improve the recurrence-free survival (RFS)^16^, ^17^, ^18^ and distant metastasis-free survival^19^ in patients with completely resected stage III cutaneous melanoma. In three randomized, controlled trials, 1-year adjuvant treatment with ICIs (nivolumab or pembrolizumab) or TT (combination dabrafenib-trametinib) was compared with placebo after complete resection in patients with stage IIIA-C melanoma. In patients with stage N1a melanoma, tumor load in the sentinel node had to be >1 mm in greatest diameter, according to the Rotterdam criteria.^20^, ^21^, ^22^ As compared with routine surveillance, adjuvant systemic therapy improved 3-year RFS from 39% to 58% (placebo versus dabrafenib-trametinib), 45%-58% (ipilimumab versus nivolumab), and 44%-64% (placebo versus pembrolizumab).^23^, ^24^, ^25^ Although the OS data of the pivotal trials were still awaited,^16^^,^^18^^,^^26^ the Food and Drug Administration (FDA) and European Medicines Agency (EMA) have already granted extension to adjuvant treatment with dabrafenib-trametinib, nivolumab, pembrolizumab, and ipilimumab (FDA only). In clinical practice, adjuvant treatment is usually selected based on melanoma characteristics and the inclusion criteria of these phase III trials. Most patients with completely resected stage III *BRAF*-mt cutaneous melanoma are candidates for any of the three different adjuvant treatments, whereas patients with completely resected stage III *BRAF* wild-type melanoma are only candidates for adjuvant treatment with pembrolizumab or nivolumab. The preferred adjuvant systemic treatment strategy has, however, not yet been defined.

The number of patients with resected high-risk stage III melanoma treated in the adjuvant setting is still increasing, placing a significant burden on health care budgets. To compare the efficacy of the three EMA-approved adjuvant systemic treatments (nivolumab, pembrolizumab, and dabrafenib-trametinib) with placebo (as a proxy for routine surveillance) and to evaluate their socioeconomic impact, a cost-effectiveness analysis (CEA) was carried out.

## Materials and methods

### Model resources

For this CEA model, clinical data on drugs that have been approved by EMA for adjuvant treatment of resected high-risk melanoma were used. These (updated) data were obtained from the three pivotal phase III trials: CheckMate 238 (nivolumab), KEYNOTE-054 (pembrolizumab), and COMBI-AD (dabrafenib-trametinib).^16^^,^^18^^,^^23^^,^^25^ As compared with KEYNOTE-054 and COMBI-AD, CheckMate 238 was different regarding the comparator arm (i.e. ipilimumab instead of placebo) and patient population (exclusion of resected stage IIIA and inclusion of resected stage IV). Routine surveillance is usually applied to patients who do not have adjuvant treatment because of co-morbidities or patients’ wishes. In addition, EMA has not granted approval for ipilimumab in the adjuvant setting because of severe adverse events (AEs).^26^ Therefore, placebo (as a proxy for routine surveillance) was used as comparator for the three adjuvant treatment strategies with nivolumab, pembrolizumab, and dabrafenib-trametinib.

### Model structure

**Figure 1:**
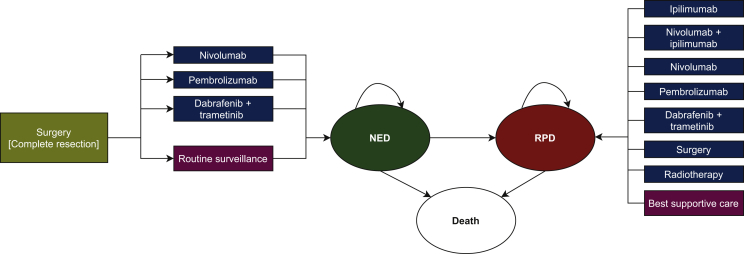
**Markov model depicting different treatment strategies.** After complete surgical resection of stage III melanoma, patients could either receive adjuvant treatment (nivolumab, pembrolizumab, or dabrafenib-trametinib) or routine surveillance. The model was based on three health states: NED, RPD, and death. In patients with RPD, different treatment strategies could be applied (i.e. systemic versus locoregional treatment or best supportive care). NED, no evidence of disease; RPD, recurrent/progressive disease.

To compare these three adjuvant systemic treatment strategies (nivolumab, pembrolizumab, and dabrafenib-trametinib) with routine surveillance (placebo) in patients with resected stage III (lymph node metastasis >1 mm) melanoma, a computer-based (Microsoft Excel®, Microsoft Corp., Redmond, WA) interactive Markov model has been developed (Figure 1). Although no direct comparison data of nivolumab and pembrolizumab were available, these ICIs were considered interchangeable, and the Kaplan–Meier (KM) curves for patients treated with pembrolizumab in the adjuvant setting were also used for patients treated with nivolumab.

The model consisted of three health states: no evidence of disease (NED), recurrent/progressive disease (RPD), and death. Since the long-term RFS and OS data are not available yet, extrapolation beyond the clinical trial data was carried out. The relevant values on the x-axis and y-axis from the corresponding KM curves were extracted using Plot Digitizer v2.6.9.^27^ The Hoyle and Henley method^28^ was used to conduct parametric survival modelling, first in Microsoft Excel® and then using the survival package in R. The following distributions were estimated: exponential, Weibull, log-normal, and log-logistic.

To conduct a CEA from societal perspective, both the cost measures and well-being (utility) were taken into account;^29^ model outcomes included all relevant costs (in 2020 €), life years (LYs) and quality-adjusted life years (QALYs). To compare the cost-effectiveness of different adjuvant treatment strategies, we calculated the incremental cost-effectiveness ratios (ICERs); the difference in costs divided by the difference in LYs and in QALYs. As survival data were (more) immature of the adjuvant trial with pembrolizumab, data from the extrapolated OS KM curve of the adjuvant trial with dabrafenib-trametinib were used.

### Model transitions and survival estimates

**Figure 2:**
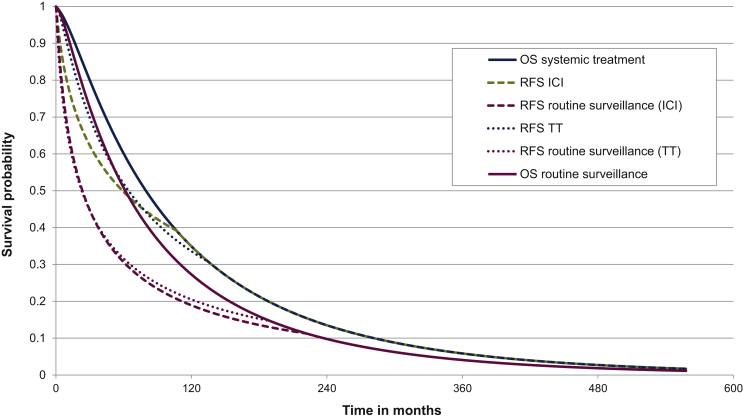
**Modelled Kaplan–Meier curves.** Modelled RFS curves of pembrolizumab (also used for nivolumab, ICI), dabrafenib-trametinib (TT), and routine surveillance were based on corresponding trials, i.e. KEYNOTE-054 and COMBI-AD, respectively. In the absence of OS data, RFS data were extrapolated (lifetime horizon) and corrected for background mortality. As the RFS data from the COMBI-AD trial were the most mature, these OS data were used for all adjuvant systemic treatment regimens (dabrafenib-trametinib, as well as pembrolizumab and nivolumab) versus placebo (as a proxy for routine surveillance). ICI, immune checkpoint inhibitor (nivolumab/pembrolizumab); OS, overall survival; RFS, recurrence-free survival; TT, targeted therapy (dabrafenib-trametinib).

Transitions between health states (NED-RPD-death) corresponded with the time interval between drug administration (2 weeks for nivolumab, 3 weeks for pembrolizumab, and 4 weeks for dabrafenib-trametinib).^16^, ^17^, ^18^ The scanning intervals were 3-monthly during the first 2 years and 6-monthly in the 3 years thereafter. (Dis)continuation probabilities could be calculated using data from the extrapolated (RFS and OS) KM curves^23^^,^^25^ (Figure 2). For example, in the pembrolizumab model, which was also applied for nivolumab, approximately 75% of patients were recurrence-free during the first year, whereas 62% of patients were recurrence-free in the routine surveillance group. The extrapolated OS data were corrected for background mortality (mortality due to other causes than melanoma).^30^ To estimate the best fitted KM curve, Akaike’s information criterion value was used in addition to visual assessment. Eventually, log-normal was applied to both the RFS curves and the OS curves.

In the model, every patient started in the NED state and patients could remain disease-free and continue therapy (up to 1 year) or experience recurrence. Once a patient had recurrence, the patient could only stay in the RPD state or migrate to the ‘death’ state in the model.^31^ In case of RPD, patients could receive subsequent treatment (with accompanied costs) in terms of surgery, radiotherapy, systemic treatment, or a mix of these (Figure 1). If necessary, patients could receive best supportive care (BSC), including palliative care. An overview of the (rate of) subsequent systemic treatments were derived from the (limited) available data and is illustrated in Supplementary Figure S1, available at https://doi.org/10.1016/j.esmoop.2021.100303.^32^, ^33^, ^34^ Grade 3 or 4 AEs could also delay or result in discontinuation of adjuvant treatment. Since none of the grade 3 or 4 AE categories exceeded the 5% incidence threshold (observed in the associated clinical trials^16^, ^17^, ^18^), AEs, both in disutility and costs, were not taken into account. Therefore, it was assumed that AEs would have a minor impact on the results.

### Utilities and costs

The QALY captures gains from reduced morbidity (quality gains) and reduced mortality (quantity gains), and combines these gains into a single measure. QALYs are calculated by multiplying the amount of LYs gained by a utility value [i.e. an indicator for the quality of life (QoL), which varies between zero (death) and one (perfect health)].^35^ Data on clinical outcomes, utility values, and QoL were obtained from the phase III trials and observational datasets (e.g. administrative registries and literature, Table 1). Within costs, a distinction was made between health care costs and societal costs.

**Table 1:** Base-case cost-effectiveness results for adjuvant treatment (compared with routine surveillance)

	Immune checkpoint inhibitors			Targeted therapy	
	Nivolumab	Pembrolizumab	Routine surveillance	Dabrafenib-trametinib	Routine surveillance
Total costs (health care + societal)	168 826	194 529	126 096	211 110	134 945
Health care costs per patient					
Adjuvant treatment costs	64 094	89 920	—	108 295	—
Drug acquisition costs	60 730	87 672	—	108 283	—
Drug administration costs	3364	2248	—	12	—
Subsequent treatment costs	72 282	72 282	76 522	75 537	87 378
Drug acquisition costs	71 262	71 262	75 342	74 530	86 310
Drug administration costs	1020	1020	1180	1007	1068
Health care costs	15 421	15 298	15 769	15 711	14 802
NED	3591	3468	3 073	4942	3052
RPD	9222	9222	9 933	8161	8986
BSC	2608	2608	2 763	2608	2764
Societal costs per patient					
Societal costs	17 029	17 029	33 804	11 567	32 764
Informal care	3862	3862	8422	2557	7943
Productivity	12 987	12 987	25 333	8820	24 777
Travel	180	180	49	190	44
Utilities					
QALY	7.43		5.41	7.46	5.43
NED	6.78		4.05	7.03	4.15
RPD	0.64		1.36	0.43	1.28
LYs	8.70		7.29	8.70	7.29
NED	7.82		5.28	8.10	5.42
RPD	0.88		2.01	0.60	1.87
ICER					
Per QALY	21 153	33 878	—	37 520	—
Per LY	30 305	48 543	—	54 018	—

The base case is the expected case of the model, determined by the various input parameters and assumptions, in € or utilities.

BSC, best supportive care (including palliative care); ICER, incremental cost-effectiveness ratio; LY, life year; NED, no evidence of disease; QALY, quality adjusted life year; RPD recurrent/progressive disease.

Drug costs (flat dose) for adjuvant systemic treatment were taken from a Dutch database for drug costs^36^ and were translated into 2-week cycles (nivolumab), 3-week cycles (pembrolizumab), and 4-week cycles (dabrafenib-trametinib) as were applied in the registration trials. Drug costs were multiplied with the probability and the duration of the treatment and the number of patients receiving systemic treatment, using the formula *RFS (t-1) – RFS(t)*. Supplementary Tables S1 and S2, available at https://doi.org/10.1016/j.esmoop.2021.100303, outline the model input parameters and its resources. The costs were adjusted to 2020 prices using the general price index from the Dutch Central Agency for Statistics.^37^ The base case of the model assumed flat dosing regimens for all therapeutic drugs, except for ipilimumab (RPD setting), for which a median weight of 78.4 kg was applied.^38^ All future costs were discounted by 4.0% and future health benefits by 1.5%, over a lifetime horizon (with a median patient age of 50 years), as prescribed by the Dutch guidelines for conducting economic evaluations in health care.^39^

### Sensitivity analysis

**Figure 3:**
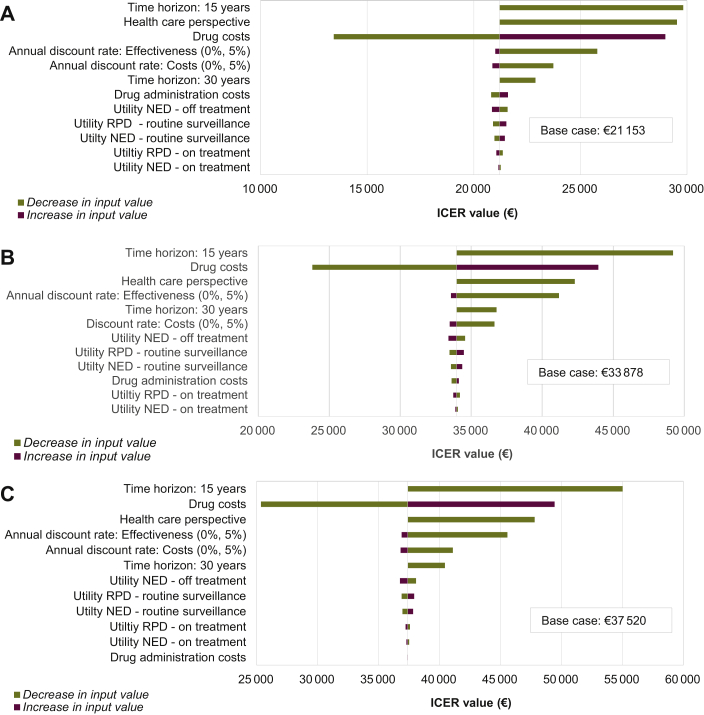
**Sensitivity analyses—Tornado diagrams.** Tornado diagrams for deterministic sensitivity analysis depicting results of changes in selected variables per adjuvant systemic treatment therapy. (A) Nivolumab versus routine surveillance. (B) Pembrolizumab versus routine surveillance. (C) Dabrafenib-trametinib versus routine surveillance. Each bar represents a range of expected decrease (blue) or increase (red) in value per variable. Results are presented in ICERs. ICER, incremental cost-effectiveness ratio; NED, no evidence of disease; RPD, recurrent/progressive disease.

To determine the influence of input parameter uncertainty (mean and variance) and the uncertainty arising from methodological assumptions made throughout the construction of the model, sensitivity analyses (both deterministic and probabilistic) were used. Evaluating the robustness of the results was carried out according to decision analytic modeling.^31^^,^^40^ In the deterministic sensitivity analysis (DSA), input parameters were varied one at a time in Tornado diagrams (Figure 3). For probabilistic sensitivity analyses (PSA), variations in multiple parameters at the same time were taken into account, by performing 1000 Monte-Carlo simulations.^31^ Samples were randomly drawn from the distributions (ɣ-distribution for costs, β-distribution for utility values) for all parameters at once using Microsoft Excel® (Supplementary Table S1, available at https://doi.org/10.1016/j.esmoop.2021.100303). Utilities were varied over their 95% confidence intervals, costs within 25% of their baseline values.

To better understand the clinical application of these calculations, cost-effectiveness acceptability curves (CEACs) were generated, using a €50 000 willingness to pay (WTP) threshold. WTP is a preference-based construct that reflects burden of disease (describing loss of health and death due to disease^41^) by assessing the monetary value of a hypothetical cure for a disease.^42^ After surgical complete resection, patients have NED when adjuvant treatment is started and the burden of disease is considered intermediate.^43^ In contrast to advanced/metastatic melanoma, that carries a high burden of disease and is accompanied with a WTP of €80 000 per QALY gained, WTP for patients with resected stage III melanoma has been set at €50 000 per QALY gained (in the Netherlands).^43^

## Results

### Base-case results

Based on the modeled RFS and OS curves, total costs were €168 826 for nivolumab, €194 529 for pembrolizumab, and €211 110 for dabrafenib-trametinib, whereas costs for routine surveillance varied from €126 096 to €134 945 (for nivolumab/pembrolizumab versus dabrafenib-trametinib, respectively). An overview of the model input parameters and base-case results of the CEA of each postoperative treatment regimen is provided in Supplementary Tables S1 and S2, available at https://doi.org/10.1016/j.esmoop.2021.100303, respectively. Drug acquisition costs placed a significant burden on the health care budget (per patient): €64 094 for nivolumab, €89 920 for pembrolizumab, and €108 295 for dabrafenib-trametinib.

Societal costs ranged from €11 567 to €33 804, of which approximately €12 000 could be attributed to lost productivity costs for patients who were treated with systemic therapy in the adjuvant setting and €25 000 for the routine surveillance group. Over a lifetime, patients gained 1.41 LYs and 2.02 QALYs when treated with ICIs (nivolumab/pembrolizumab) compared with routine surveillance. For patients treated with dabrafenib-trametinib, the gained LYs and QALYs were 1.41 and 2.03, respectively. In terms of incremental costs and benefits, ICERs were €21 153/QALY and €30 305/LY for nivolumab, €33 878/QALY and €48 534/LY for pembrolizumab, and €37 520/QALY and €54 018/LY for dabrafenib-trametinib, when compared with routine surveillance.

### Sensitivity analysis

#### DSA

The DSA showed robust base-case results. In Figure 3, the Tornado diagrams reflect the 12 sensitivity analyses which had the most influence on the ICER when comparing adjuvant treatment with routine surveillance. Changes in time horizon influenced all ICERs. The utility values had almost no impact on the ICERs of ICIs (nivolumab and pembrolizumab), whereas for TT (dabrafenib-trametinib) administration costs had the least impact on ICER.

#### PSA

**Figure 4:**
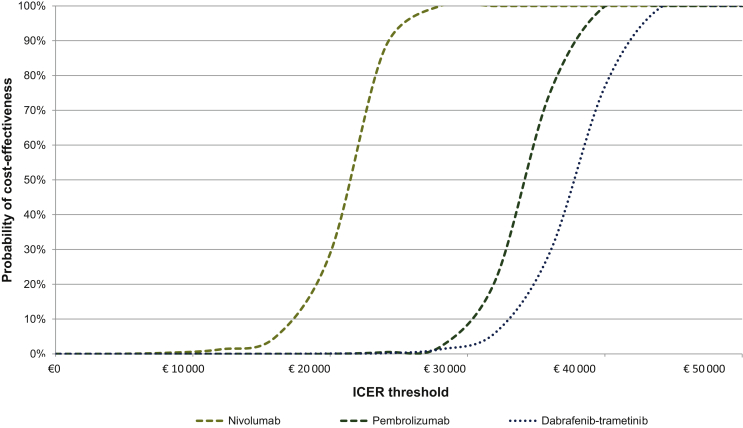
**Sensitivity analyses—cost-effectiveness acceptability curves.** These curves summarize information on uncertainty in cost-effectiveness estimates for nivolumab, pembrolizumab, and dabrafenib-trametinib. For patients with resected high-risk stage III melanoma, the willingness to pay (WTP) threshold is €50 000. ICER, incremental cost-effectiveness ratio; WTP, willingness to pay.

Figure 4 shows the PSA reflected in CEACs. Using a WTP threshold of €50 000/QALY gained, the probability of adjuvant systemic treatment (pembrolizumab, nivolumab, or dabrafenib-trametinib) being cost-effective was 100%.

## Discussion

This study provides a comprehensive CEA of the three EMA-approved adjuvant systemic treatments in patients with completely resected high-risk stage III melanoma. According to our Markov model, adjuvant treatment with nivolumab is the most profitable in terms of ICER (€21 153/QALY), followed by pembrolizumab (€33 878/QALY). Combination therapy with dabrafenib-trametinib was the least cost-effective (€37 520/QALY). This difference can largely be attributed to the high drug acquisition costs for adjuvant treatment, ranging from €64 094 (nivolumab) to €89 920 (pembrolizumab), to €108 295 (dabrafenib-trametinib) per patient. Compared with QALYs in patients with routine surveillance (5.41-5.43), QALYs improved for patients with adjuvant treatment with pembrolizumab or nivolumab (7.43) and dabrafenib-trametinib (7.46), while gaining 1.41 LYs (all systemic therapies). Changing the time horizon from lifetime to 15 years yielded the greatest effect on the model results, which indicates that most costs are accumulated at the beginning of the treatment.

One of the challenges was to compare the three adjuvant trials since eligibility criteria and comparator arms were different; the KEYNOTE-054 and COMBI-AD included patients with resected stage IIIA-IIIC melanoma and had placebo in the comparator arms, whereas CheckMate 238 included patients with resected stage IIIB-IV melanoma and had ipilimumab as comparator instead of placebo.^16^, ^17^, ^18^ Using the RFS for these different disease stages would have led to a skewed comparison in terms of extrapolated RFS and OS, as patients with resected stage IV melanoma have a worse prognosis.^44^^,^^45^ Therefore, the RFS rates for patients treated with pembrolizumab were also applied for patients treated with nivolumab. Although KEYNOTE-054 did not include patients with in-transit metastases, the distribution of melanoma stages was comparable with the COMBI-AD trial (16% versus 19% stage IIIA; 46% versus 39% stage IIIB; 38% versus 41% stage IIIC for pembrolizumab and dabrafenib-trametinib, respectively), as well as median age (54 versus 50 years).

When comparing the effectiveness of ICIs (pembrolizumab, nivolumab) with TT (dabrafenib-trametinib) in patients with resected stage III melanoma, the 1-year RFS rate was higher for dabrafenib-trametinib (75% versus 88%), whereas the 3-year RFS rate appeared to be higher in patients treated with ICIs (64% versus 58%). This resulted in a favorable extrapolation of the RFS curve for ICIs, which is in line with the clinical observations of TT in patients with metastatic melanoma. TT is known for rapid tumor response, but also for rebound tumor progression after discontinuation of treatment.^13^ If RFS would be assumed as a surrogate for OS^46^ and taking into account the favorable 3-year RFS rate of ICIs, ICER would further increase in favor of ICIs.

In this CEA, adjuvant treatment strategies of stage III melanoma were compared according to all relevant utilities and costs, including health care and societal costs. Our model shows that cost-effectiveness mainly depends on drug acquisition costs, which is in line with other Markov models comparing systemic adjuvant therapies (including ipilimumab) from a US ($) perspective.^47^^,^^48^ Although discrepancies between formal and actual list prices are not uncommon, drug prices seemed to be quite similar in The Netherlands.^49^ The actual prices are not freely accessible, however, and are different across countries. In contrast to the phase III trials in which weight-based dosing was used, we assumed a flat-fixed dosing regimen for all patients, as this is widely applied in current clinical practice and no patient-level data were available. Furthermore, the optimal drug dosing (weight-based versus fixed-flat dosing) is open for debate.^50^, ^51^, ^52^ Other health care costs were related to hospital visits (including drug administration and admissions), diagnostics, local treatment (radiotherapy/surgery), or BSC. Since dabrafenib-trametinib is not administered intravenously at the outpatient clinic, but can be taken orally at home, drug administration costs were negligible. The model incorporated the scanning and treatment intervals of the adjuvant trials (2-weekly for nivolumab, 3-weekly for pembrolizumab, and 4-weekly for dabrafenib-trametinib). In current clinical practice, longer time intervals between treatments (i.e. 4-weekly for nivolumab and 6-weekly for pembrolizumab) are increasingly common, especially during the current COVID-19 pandemic, where hospital visits are limited as much as possible. While the frequency of hospital visits will have limited impact on actual costs, extending these intervals could reduce the burden on both patients and health care resources, and could be a first step to reduce costs. Furthermore, the adjuvant treatment duration is currently set at 1 year. To reduce treatment-related toxicity while limiting drug spillage and costs, the optimal adjuvant treatment strategy should be determined. The optimal duration of treatment in patients with advanced melanoma is already being investigated in a prospective trial.^53^

The model input had several limitations worth mentioning. First, since adjuvant treatment of melanoma has been introduced in clinic recently, long-term real-world data on efficacy (i.e. RFS and OS) and QALYs are not available yet. In addition, the clinical trial data did not provide detailed information on type of recurrence and subsequent treatment. Therefore, assumptions had to be based on the current available (trial) data which may not reflect the real-world outcomes.^54^ Second, according to traditional methods to perform a CEA of anticancer drugs using a Markov model, patients’ health states can be stable or worsen, but never improve (i.e. from RPD to NED). In real life, patients’ health states can improve, since patients can transit to NED. As the QALYs capture decrease in QoL after disease progression, this might have resulted in an underestimation of QALYs. This limitation, however, was applicable for all three adjuvant treatment strategies. Third, according to CEA guidelines, AEs were not included as no grade 3-4 AEs exceeded the threshold of 5% incidence.^16^, ^17^, ^18^ Whereas the impact of AEs on ICERs is considered to be limited, patients suffer from these AEs, which can be lifelong and sometimes require lifelong therapy such as hormone replacement.^55^ In addition, AEs in patients treated with TT (dabrafenib-trametinib) are more often reversible,^56^ which could result in lower costs for supportive care.

When data of patients with stage III melanoma grow more mature, it would be useful to ascertain these assumptions with real-world data. Therefore, the Dutch Melanoma Treatment Registry has started to collect real-world data of patients with stage III melanoma since 2019 in the Netherlands.^57^ This registry has shown that the number of patients with melanoma who are treated with (adjuvant) systemic therapy is increasing every year. In addition, since the first results of an ongoing randomized trial in patients with high-risk stage II melanoma showed prolonged RFS in patients treated with adjuvant pembrolizumab,^58^ it is expected that these patients become candidates for adjuvant treatment in the (near) future.

To reduce costs by preventing over-treatment with adjuvant therapy, (bio)markers are urgently needed to identify patients who are not at risk of recurrent disease. Effective prognostic markers could assist clinical decision making to spare patients adjuvant treatment and treatment-related AEs, which could be lifelong. Negotiating drug acquisition costs to further reduce the burden on health care resources is inevitable, especially given the increasing number of patients treated with adjuvant systemic therapy, as well as the advent of new expensive drugs for other indications. In addition, it may be helpful to favor longer treatment intervals and to evaluate shorter treatment duration (<1 year) of adjuvant systemic drugs.

In conclusion, this is the first CEA comparing the three EMA-approved adjuvant treatment strategies of patients with resected high-risk stage III melanoma, showing beneficial outcomes in terms of (short-term) RFS and QALYs for pembrolizumab, nivolumab, and dabrafenib-trametinib compared with routine surveillance. Though all three evaluated treatments are below the WTP threshold of €50 000/QALY gained, high drug costs place a significant burden on the health care budget. Since an increasing number of patients with melanoma are being treated with adjuvant systemic therapy, the socioeconomic impact needs to be reduced by clinical use of effective prognostic tools, less intensive treatment regimens (dosing, interval, and treatment duration), and a significant reduction in drug acquisition costs.

## Supplementary Data

Appendix Tables

Figure 1 (A) Treatment regimen. (B) Treatment regimen and percentages.
