# Supplementary material for: Cost-effectiveness of adjuvant systemic therapies for patients with high-risk melanoma in Europe: a model-based economic evaluation
Source: ESMO Open. 2021 Nov 13;6(6):100303. doi: 10.1016/j.esmoop.2021.100303 (PMC8599106; doi:10.1016/j.esmoop.2021.100303)
Supplement: Appendix Tables [file mmc1.docx]

# **Appendix**

Table A.1. Model input parameters

1. **Unit costs used for the Markov model**
   *CT, computed tomography;* ^18^F-FDG PET/CT, *^18^F-fluorodeoxyglucose positron emission tomography/CT; MRI, magnetic resonance imaging.*

| **Drug acquisition costs,** *Dutch online drug prices (2019)* | | | | | |
| --- | --- | --- | --- | --- | --- |
| *Drug* | *Dose (mg)* | *Price tablet/vial (€)* | *Dosing schedule (mg/interval)* | *Weighted cost per dose (€)* | *Administration route* |
| Nivolumab | 40 | 441.48 | 240mg / 2 weeks | 2,648.86 | Intravenous |
|  | 100 | 1,103.69 |  |  |  |
| Pembrolizumab | 100 | 2,860.56 | 200mg / 3 weeks | 5,721.21 | Intravenous |
| Dabrafenib | 50 | 38.73 | 300mg / day | 113.7 | Oral |
| Trametinib | 0.5 | 59.06 | 2mg / day | 222.15 | Oral |
| Ipilimumab | 50 | 4,632.50 | 250mg / 3 weeks | 23,162.50 | Intravenous |
| **Drug administration costs** | | | | | |
| *Route* | *Dosing* | | *Costs (€)* | *Reference* | |
| Intravenous | Every cycle | | 146.72 | *Franken et al. (2018)* | |
| Oral | Once | | 12.00 | *Dutch costing manual (2016)* | |
| **Costs regarding other health care use** | | | | | |
| *Type of Service* | | | *Costs (€)* | *Reference* | |
| Visit outpatient dermatology clinic | | | 98.41 | *Dutch costing manual (2016)* | |
| Visit outpatient oncology clinic | | | 142.75 | *Dutch costing manual (2016)* | |
| Visit outpatient surgery clinic | | | 78.95 | *Dutch costing manual (2016)* | |
| MRI of brain | | | 222.78 | *Dutch costing manual (2016)* | |
| Complete blood count | | | 31.43 | *Dutch costing manual (2016)* | |
| CT | | | 177.44 | *Franken et al. (2016)* | |
| ^18^F-FDG PET/CT | | | 1000.84 | *Franken et al. (2016)* | |
| Progression resource costs | without systemic treatment | | 8,302.23 | *Leeneman et al. (2020)* | |
|  | with systemic treatment | | 17,311.21 | *Leeneman et al. (2020)* | |
| End-of-life costs | | | 3,650.83 | *NICE TA553 (2018)* | |
| **Societal costs,** *Dutch costing manual (2016)* | | | | | |
|  | | | | *Unit* | *Standard values* |
| **Transportation** | Average distance (from patients’ home to hospital) | | | km | 7 |
|  | Car, cost per kilometer | | | € | 0.19 |
|  | Car, (average) parking cost | | | € | 3.00 |
|  | Public transport, cost per kilometer | | | € | 0.19 |
| **Productivity costs & informal care** | Paid work | Friction period | | days | 107 |
|  |  | Productivity costs: hourly wage (general) adjusted for inflation | | € | 34.75 |
|  | Unpaid work | Replacement costs unpaid work adjusted for inflation | | € | 14.00 |

**B. Base case input parameters, including utilities and costs**

*NED, no evidence of disease; RPD, recurrent/progressive disease; SE, standard error.*

| **Costs (€)** | | | | | |
| --- | --- | --- | --- | --- | --- |
|  | *Parameters* | *Mean* | *SE* | *Distribution* | *Source* |
| Drug acquisition costs | Nivolumab | 2,648.86 | +/- 25% | γ | *Dutch online drug prices (2019)* |
|  | Pembrolizumab | 5,721.21 | +/- 25% | γ | *Dutch online drug prices (2019)* |
|  | Dabrafenib- trametinib | 9,440.55 | +/- 25% | γ | *Dutch online drug prices (2019)* |
| Administration costs | | 146.72 | +/- 25% | γ | *Dutch costing manual (2016)* |
| **Utilities and side effects** | | | | | |
|  | *Parameters* | *Mean* | *SE* | *Distribution* | *Source* |
| ***Adjuvant therapy*** | | | | | |
| NED | On treatment | 0.854 | 0.006 | ß | *NICE (2018)* |
|  | Off treatment | 0.869 | 0.005 | ß | *NICE (2018)* |
| RPD |  | 0.730 | 0.016 | ß | *NICE (2018)* |
| ***Routine surveillance*** | | | | | |
| NED |  | 0.869 | 0.005 | ß | *NICE (2018)* |
| RPD |  | 0.730 | 0.016 | ß | *NICE (2018)* |

## Figure A.1. Subsequent treatments and percentages

1. **Flowchart of subsequent treatment after complete surgical resection.** For patients with adjuvant systemic treatment and patients with routine surveillance.

*NED, no evidence of disease; RPD, recurrent/progressive disease*

1. **Table showing corresponding percentages per scenario.**
