# Supplementary figures and images for: Cost-effectiveness of adjuvant systemic therapies for patients with high-risk melanoma in Europe: a model-based economic evaluation
Source: ESMO Open. 2021 Nov 13;6(6):100303. doi: 10.1016/j.esmoop.2021.100303 (PMC8599106; doi:10.1016/j.esmoop.2021.100303)

A.

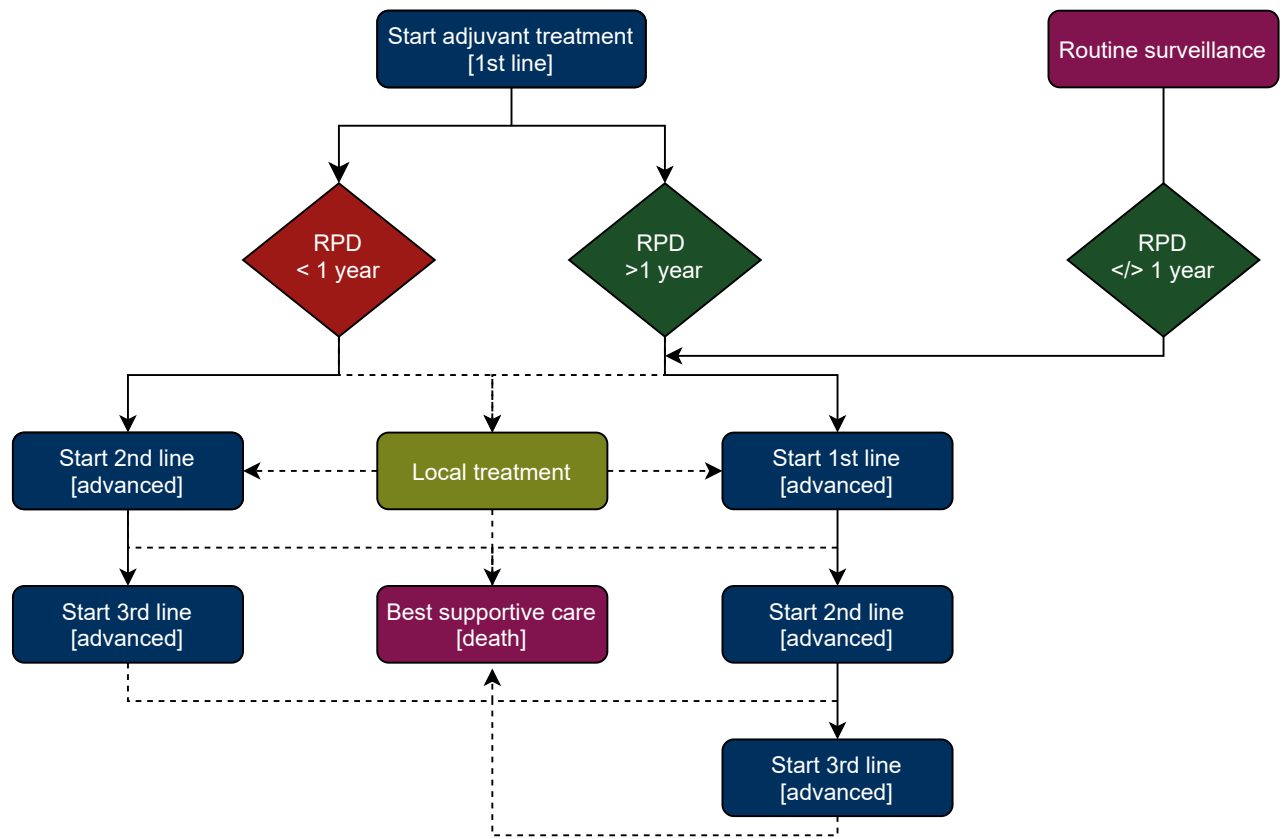

**B.**

[illegible]

Supplement: Figure 1 — (A) Treatment regimen. (B) Treatment regimen and percentages. [file mmc2.pdf]
